# Facilitating informed decisions about breast cancer screening: development and evaluation of a web-based decision aid for women in their 40s

**DOI:** 10.1186/s12911-017-0423-7

**Published:** 2017-03-21

**Authors:** Elena B. Elkin, Valerie H. Pocus, Alvin I. Mushlin, Tessa Cigler, Coral L. Atoria, Margaret M. Polaneczky

**Affiliations:** 1 Department of Epidemiology and Biostatistics, Memorial Sloan Kettering Cancer Center, New York, NY USA; 2 Department of Healthcare Policy and Research, Weill Cornell Medical College, New York, NY USA; 3 Department of Medicine, Weill Cornell Medical College, New York, NY USA; 4 Department of Obstetrics and Gynecology, Weill Cornell Medical College, New York, NY USA

**Keywords:** Breast cancer, Screening, Mammogram, Decision aid, Shared decision making

## Abstract

**Background:**

Expert groups and national guidelines recommend individualized decision making about screening mammography for women in their 40s at low-to-average risk of breast cancer. We created *Breast Screening Decisions* (*BSD*), a personalized, web-based decision aid, to help women decide when to start and how often to have routine screening mammograms. We evaluated *BSD* in a large, prospective pilot trial of women and their clinicians.

**Methods:**

Women ages 40–49 were invited to use *BSD* before a scheduled preventive care visit. One month post-visit, users were asked about decisional conflict, knowledge, perceptions and worry about breast cancer and screening. They were also asked whether they had a screening mammogram since their visit, scheduled an appointment for a screening mammogram, or if they were planning to schedule an appointment within the next six months. Women who responded “no” to each of these successive questions were considered to have no plan for a screening mammogram within the next 6 months, unless they explicitly stated that they were unsure about screening mammography. Clinicians were surveyed regarding mammography discussions and perceived patient knowledge and anxiety.

**Results:**

Of 1,100 women invited to use *BSD*, 253 accessed the website, and 168 were eligible to participate in the pilot study. One-fifth had a family history of breast cancer, and at least 76% had any prior mammogram. At follow-up, 88% of *BSD* users reported discussing mammography at their visit, and 77% said they had a screening mammogram since the visit or that they made or were planning to make a screening mammogram appointment. The average decisional conflict score was 22.5, within the threshold for implementing decisions. Decisional conflict scores were lowest in women who said that they had or planned to have a mammogram (mean 21.4, 95% CI 18.3-24.6), higher in those who did not (mean 24.8, 95% CI 19.2-30.5), and highest in those who were unsure (mean 31.5, 95% CI 13.9-49.1). Most *BSD* users expressed accurate perceptions of their breast cancer risk and the benefits and limitations of screening.

**Conclusions:**

A web-based decision aid may support informed, individualized decisions about screening mammography and facilitate discussions about screening between women in their 40s and their clinicians.

**Electronic supplementary material:**

The online version of this article (doi:10.1186/s12911-017-0423-7) contains supplementary material, which is available to authorized users.

## Background

Screening mammography reduces breast cancer mortality by about 15% in women ages 40–49 [1], somewhat less than the 20–30% mortality reduction in women ages 50–69. Considering the balance of benefits and harms, in 2009 the US Preventive Services Taskforce (USPSTF) recommended against routine screening mammograms for women in their 40s, advising them instead to make individual decisions about screening with their physicians in the context of their personal breast cancer risk, values and preferences [2]. The USPSTF reaffirmed this recommendation in 2016 following an updated review of the evidence [3]. The American Medical Association, American College of Physicians, American Academy of Family Physicians and the American College of Preventive Medicine supported the panel’s 2009 recommendation. The American Congress of Obstetricians and Gynecologists (ACOG) and the American College of Radiology expressed opposition and recommended annual screening mammography starting at age 40. Until recently, the American Cancer Society also recommended annual screening for women 40 and older [4].

Controversy and conflicting guidelines have led to confusion among women and their clinicians about screening mammography [5–7]. We created *Breast Screening Decisions* (*BSD*), a web-based tool to help women in their 40s at low-to-average risk of breast cancer decide when to start and how often to have screening mammograms, in the context of a shared decision-making process with their clinicians. The goal of *BSD* is not necessarily to change screening behavior, but rather to help women make decisions about screening mammography that are informed and consistent with their values and preferences [8], and to serve as a foundation for discussions about screening mammography between women and their health care providers. We conducted a large pilot trial of *BSD* to assess its feasibility and acceptability in clinical practice, and to evaluate decisional conflict, knowledge and worry about breast cancer and screening mammography in *BSD* users.

## Methods

### Decision aid development

*BSD* was created using the Ottawa Decision Support Framework [9] and evidence-based recommendations for communication of health-related risk information [10]. *BSD* content was developed with input from breast oncologists and radiologists, internists and gynecologists. Prior to using *BSD* in this study, usability testing was conducted with clinicians and volunteers in the target age group, and the site was modified per their feedback. *BSD* meets fully 26 of 28 International Patient Decision Aid Standards (IPDAS) Content Criteria and 17 of 20 relevant IPDAS Development Process criteria (see Additional file 1: Table S1) [11]. Five of six criteria for internet-based decision aids were fully met and one was partially met.

The landing page of *BSD* frames the two decisions the tool is designed to address: when to start (age 40 or age 50) and how often to have (every year or every other year) routine screening mammograms. *BSD* users next complete a breast cancer risk assessment based on the Gail model [12, 13]. In addition to eliciting the Gail model risk factor information, *BSD* identifies women at increased risk due to: 1) a first-degree relative diagnosed with breast cancer before age 50 or ovarian cancer at any age; 2) a prior diagnosis of ductal carcinoma in situ or lobular carcinoma in situ; 3) a prior diagnosis of atypical ductal hyperplasia; 4) a known *BRCA1/2* mutation; or 5) a history of chest radiation. Women who report any high-risk criteria and women with a predicted five-year breast cancer risk ≥1.66% – consistent with accepted definitions of high risk [14] – may not proceed with the decision aid. Instead, they are told that based on the information they provided, they may be at greater-than-average risk of developing breast cancer, and that for women like them, routine mammography and possibly other screening tests may be recommended. They are encouraged to speak with their doctors about their breast cancer risk and screening options.

For women who report no high-risk criteria, *BSD* displays their personal predicted risk of developing breast cancer in the next 5 years. Risk information is presented using female icon arrays, percentages and numeric frequencies with explanatory text (“Of 1,000 women like you, *X* will develop breast cancer in the next 5 years”).

In subsequent pages, *BSD* provides information about the accuracy of screening mammograms and the benefits and harms of routine screening. Mammogram accuracy is expressed with icon arrays and frequencies to describe test results and true cancer status in 1,000 women in their 40s who have a screening mammogram, based on published estimates from the Breast Cancer Surveillance Consortium [1, 15].

Quantitatively, benefit is described as reduction in the number of deaths from breast cancer associated with the four different screening schedules reflecting different starting ages (40 vs. 50) and screening intervals (every year vs. every other year). This information, based on published estimates from the Cancer Intervention and Surveillance Modeling Network [16], is displayed using female icon arrays and frequencies with explanatory text. Differences in breast cancer death rates are estimated as a function of the relative risk reduction associated with each screening schedule and the underlying breast cancer mortality risk for women ages 40–49 [1].

The potential harms of routine screening described in *BSD* include diagnostic work-ups associated with false-positive results; overdiagnosis and unnecessary treatment; delayed cancer diagnosis and false reassurance associated with false-negative results; and cumulative radiation exposure associated with repeat screening, which is described as “extremely low.” False-positive and false-negative rates are given quantitatively. Other harms are described qualitatively and in plain language, with links to additional information about these outcomes.

Following the information about benefits and harms of screening, *BSD* users can explore their attitudes toward breast cancer, screening and involvement in health care decisions in a values-clarification exercise. Users are asked to consider a series of 10 statements and indicate their level of agreement or disagreement with each statement. Finally, *BSD* provides a one-page summary of the session, with options to save and print the summary document, which users are encouraged to share with their clinician.

A public-use version of *BSD* is available at www.breastscreeningdecisions.com.

### Decision aid evaluation

#### Subjects and recruitment

*BSD* was evaluated in a prospective, single-arm trial at a large, urban, academic medical center between March 2013 and April 2014. The study was approved by the Institutional Review Board at Weill Cornell Medical College.

We identified women ages 40–49 with a scheduled appointment for routine preventive care at one of three participating primary care and gynecology practices. Electronic health records (EHRs) were screened to exclude women with a personal history of breast cancer and women at increased risk of breast cancer due to one of the five aforementioned high-risk criteria. All other women were invited to participate and to use *BSD* at their convenience before their scheduled visit. Invitations, mailed and emailed 4–6 weeks before the scheduled visit, included a personalized letter signed by the woman’s physician with instructions for accessing the *BSD* website and a unique username and password. On-site access to *BSD* at each participating clinic was offered to women who did not have internet access elsewhere.

Informed consent was obtained immediately after logging into the *BSD* website. Women could log in more than once and resume or repeat a session any time prior to their scheduled visit. Women who logged in, gave consent, met eligibility criteria and used the decision aid (*BSD* users) received follow-up as described below. There was no active follow-up with women who were invited to use *BSD* but did not log in and give consent prior to their scheduled visit (non-users), although information in the EHR about their age and mammography history was collected for eligibility screening and comparison with *BSD* users.

#### Assessments

At the conclusion of the *BSD* session, users completed an online survey about their impressions of the site, with seven statements about ease of navigation, clarity and importance of information, usefulness of graphs and figures, time burden, and whether they would recommend the site to other women. Responses to each statement were given on a 5-point scale from “strongly agree” to “strongly disagree.”

One month after each user’s scheduled preventive care visit, she was contacted by telephone and asked to complete a follow-up survey by phone or online. In order to identify potential unintended impacts of the decision aid on behavior, actual or intended use of screening mammography was ascertained by asking women whether they had a screening mammogram since their visit, scheduled an appointment for a screening mammogram, or if they were planning to schedule an appointment within the next six months. Women who responded “no” to each of these successive questions were considered to have no plan for a screening mammogram within the next 6 months, unless they explicitly stated that they were unsure about screening mammography in response to the final question. Decisional conflict was assessed using O'Connor’s Decisional Conflict Scale (DCS), a 16-item instrument developed to evaluate health care decision support interventions [17]. The DCS has high reliability, and DCS scores below 25 have been associated with implementing decisions. Breast cancer worry was assessed using items adapted from McCaul’s Breast Cancer Worry Scale (BCWS) [18]. Subjects were asked how often they worry about breast cancer, how often worry about breast cancer affects their mood, and how often worry about breast cancer affects their performance of daily activities, with responses given on a 5-point scale from “not at all” to “almost all the time”. Knowledge and beliefs about breast cancer and screening mammography were assessed using individual items developed for this study or selected from existing instruments [19]. Information about each subject’s age, race, marital status, number of children, type of health insurance and prior use of mammography – for screening or diagnostic purposes – was obtained from the EHR.

Clinicians were asked to complete a brief paper survey at the conclusion of each woman’s scheduled preventive care visit. They were asked if screening mammography was discussed during the visit, the duration of the discussion in minutes, whether the patient seemed informed about screening mammography and whether the patient seemed anxious about mammography or breast cancer. Responses to these questions were given on a 5-point scale. Clinicians were blinded to information about each woman’s use of the decision aid, unless a woman volunteered this information during her scheduled visit. Thus, clinician assessments were conducted for both *BSD* users and non-users.

#### Analysis

All survey responses were analyzed using descriptive statistics, including frequencies, means and medians. DCS scores were estimated using that instrument’s scoring algorithm. Associations between predicted breast cancer risk and responses to selected items in the follow-up survey were assessed using one-way analysis of variance (ANOVA). Analysis of associations between between other outcomes were assessed using Fisher’s exact test. All analyses were performed in SAS (version 9.4, Cary, NC).

## Results

### Characteristics of the Sample

**Fig. 1:**
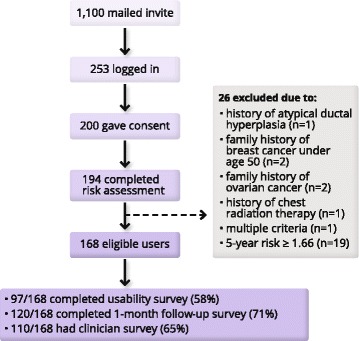
Study recruitment

From March 1, 2013 through June 30, 2014, invitations were mailed to 1,100 women ages 40–49 with a scheduled preventive care visit who had no study exclusion criteria identified in the EHR. Of these women, 253 logged in to the *BSD* website and 194 provided consent, of whom 168 met criteria for low-to-average breast cancer risk (Fig. 1). *BSD* users and non-users had the same median age (44 years). Among women with information available in the EHR, the probability of any prior mammogram (screening or diagnostic) was similar in *BSD* users (76%) and non-users (84%).

*BSD* users were predominantly white, most were married, had at least one child, and were non-smokers (Table 1). Nearly all had private, commercial health insurance. At least 24% of *BSD* users had any family history of breast cancer in someone other than a first-degree relative diagnosed at age 50 or younger, and at least 74% had a prior screening mammogram. Based on the information entered in the decision aid by users, predicted 5-year breast cancer risk varied from 0.4% to 1.6%, with a median of 1.0%. Median lifetime breast cancer risk was 12.5%.

**Table 1:** Characteristics of decision aid users

	*N*	%
Age		
40–44	101	60
45–49	67	40
Race/ethnicity		
White	135	80
Asian	20	12
Hispanic	8	5
African-American	4	2
Missing	1	<1
Married or partnered		
Yes	129	77
No	39	23
Children		
Yes	132	79
No	36	21
Smoking status		
Never smoker	134	80
Current smoker	1	<1
Former smoker	33	20
Health insurance		
Private	161	96
Public	4	2
Self-pay	3	2
Family history of breast cancer*		
Yes	40	24
No or no information	128	76
Prior screening mammogram		
Yes	125	74
No	31	18
No information	12	7
Prior diagnostic mammogram		
Yes	30	18
No	117	70
No information	21	13
Any prior mammogram		
Yes	128	76
No	29	17
No information	11	7

All information obtained from electronic health records, except for race/ethnicity, which was reported by users in the breast cancer risk assessment portion of the decision aid

*Women with a family history of breast cancer in a 1^st^-degree relative before age 50 were excluded

### Impressions of the site

At least 97 of the 168 eligible users (58%) completed the exit survey on the final page of *BSD*, and therefore viewed all prior pages. A majority of these women expressed favorable impressions of *BSD*. More than 95% agreed or strongly agreed that the site was easy to navigate, that the information was presented clearly, and that the information was important for women like them. Seventy-six percent said that graphs and illustrations helped them understand the information presented. No users said that information in *BSD* was confusing, and only 5% reported that the website was too slow. Almost 80% said they would recommend *BSD* to other women in their 40s.

### Screening decisions and decisional conflict

**Fig. 2:**
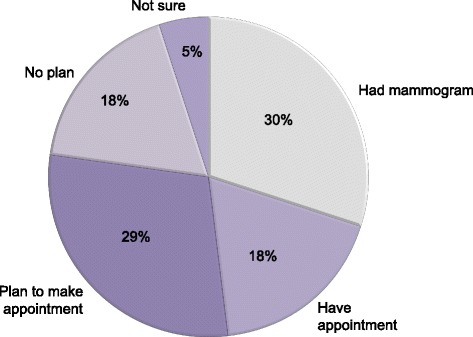
Actual and intended use of screening in *BSD* users*. *Self-reported in follow-up survey one month after scheduled preventive care visit following use of *Breast Screening Decisions* (*BSD*)

Of the 168 eligible women who gave consent and used *BSD*, 120 responded to at least one item in the follow-up survey. Of these women, 88% reported that they discussed screening mammography at their scheduled visit, and 77% said they had a screening mammogram since the visit, had scheduled an appointment for a screening mammogram, or were planning to make an appointment in the next 6 months (Fig. 2). The average decisional conflict score was 22.5, below the threshold score of 25 associated with implementing decisions [17, 20]. Decisional conflict scores were lowest among women who reported that they had or planned to have a mammogram (mean 21.4, 95% CI 18.3-24.6) higher in those who did not (mean 24.8, 95% CI 19.2-30.5), and highest in those who were unsure (mean 31.5, 95% CI 13.9-49.1) (Table 2).

**Table 2:** Decisional conflict and use of screening

	Actual or Intended Use of Screening Mammography*		
	Yes	No	Unsure
	*n = 89*	*n = 20*	*n = 6*
DCS score	21.4	24.8	31.5
95% CI	18.3–24.6	19.2–30.5	13.9–49.1

*Yes: respondent reported having mammogram since post-decision aid visit, having appointment for mammogram, or planning to schedule appointment within six months

*DCS* Decisional conflict scale

### Knowledge and worry about breast cancer and screening

*BSD* users generally expressed accurate perceptions of their own breast cancer risk and the benefits and limitations of screening (Table 3). However, 10% believed that they were more likely to get breast cancer than the average woman their age. Eighty-three percent agreed or strongly agreed that, for them, the benefits of screening outweighed the potential risks. Women who expressed this belief were considerably more likely than those who disagreed to report that they had a screening mammogram, had an appointment for screening or planned to make an appointment within the next 6 months (84% vs. 25%). Women with no plan for a screening mammogram were more likely to disagree or strongly disagree with the statement that the benefits of screening outweighed the risks, compared with *BSD* users who had or planned to have a mammogram (20% vs. 2%, *p* < 0.01 by Fisher’s exact test). Nearly all *BSD* users (96%) said that their doctor was knowledgeable about the benefits and risks of screening mammography.

**Table 3:** Knowledge, attitudes and beliefs about breast cancer risk and screening mammography

	Strongly agree or agree	Neither agree nor disagree	Strongly disagree or disagree
	%	%	%
I am more likely to get breast cancer than the average woman my age	10	20	70
My chance of developing breast cancer in the next 10 years is fairly low	70	27	3
My chance of developing breast cancer will increase as I get older	61	30	9
Screening mammography can detect all breast cancers	8	16	76
If a screening mammogram identifies an abnormality, it is definitely breast cancer	0	3	97
All abnormalities identified in a screening mammogram need to be removed	1	5	94
Having a screening mammogram will reduce my chance of dying from breast cancer	79	13	8
Having a screening mammogram will be inconvenient	38	16	46
Having a screening mammogram will be painful	35	24	41
Having a screening mammogram will help me worry less about breast cancer	70	16	14
For me, the potential benefits of a screening mammogram outweigh the potential risks	83	10	7
My doctor is knowledgeable about the benefits and risks of screening mammography	96	3	1

When asked how often they worry about breast cancer, more than one-fifth of *BSD* users said not at all, while only one user said almost all of the time. When asked how often breast cancer worry affected their mood and how often it affected their performance of daily activities, 69% and 86% of users, respectively, said not at all. However, 70% agreed or strongly agreed that having a screening mammogram would help them worry less about breast cancer.

### Role of predicted risk

There was no association between a user’s predicted breast cancer risk, based on the Gail model and given to her in *BSD*, and actual or intended use of screening. However, risk perception and worry about breast cancer varied with predicted risk. Women with higher predicted risk were more like to agree with the statement, “I am more likely to get breast cancer than the average woman my age,” while those with lower predicted risk were more likely to (correctly) disagree with this statement (*p* < 0.01 by ANOVA). Women with higher predicted risk were also more likely to report more frequent worry about breast cancer (*p* < 0.01 by ANOVA).

### Clinician perceptions

Clinician surveys were available for 110 of the 168 *BSD* users (65%) and for 618 non-users who met study eligibility criteria based on information found in the EHR. For 87% of *BSD* users and 84% of non-users, clinicians reported that the patient seemed well-informed about screening mammography. For 88% of *BSD* users and 94% of non-users, clinicians disagreed or expressed neutrality with the statement that the patient seemed anxious about screening mammography. Rates of clinician-reported mammography discussion were similar in users and non-users (90% and 92%), and for both groups the average time that clinicians reported discussing mammography was four minutes. Differences between users and non-users for these clinician-reported endpoints were not statistically significant.

## Discussion

Users of our personalized, web-based decision aid reported low levels of decisional conflict and high levels of knowledge about breast cancer risk and screening. Most discussed breast cancer screening with a physician, and their clinicians perceived them to be well informed about screening mammography.

Many women overestimate the benefits of mammography, some even holding the mistaken belief that screening can prevent breast cancer [21]. Most *BSD* users in our study (79%) agreed that having a screening mammogram would reduce their chance of dying from breast cancer, a fact stated in the decision aid. Contrary to information presented in *BSD*, 8% of users said that screening mammography can detect all breast cancers. It is not clear whether these women were unable to process or retain information presented in the decision aid, or whether their beliefs about screening mammography were too strong to counteract conflicting information.

*BSD* was designed for women at low-to-average risk of developing breast cancer, and eligible users are told that they are at low-to-average risk based on their personalized risk prediction. Despite this, 10% of *BSD* users perceived that they were “more likely to get breast cancer than the average woman” their age. Thus, for some women there is discordance between perception and information, even when breast cancer risk estimates are personalized and stated explicitly, perhaps due to lack of confidence in those estimates [22].

In the context of disease screening, decision aids have been shown to improve knowledge, reduce decisional conflict and anxiety, allow patients to be active participants in their care and lead to informed, values-based decisions [23–25]. A screening mammography decision aid for Australian women under 50 increased knowledge and reduced indecision without increasing feelings of anxiety [26], and information about “overdetection” of breast cancer further enhanced the likelihood of making an informed decision [27].

In the absence of a comparison or control group in our study, we cannot make causal inferences about *BSD* and its relationship with decision-making outcomes. *BSD* users were a self-selected group, and their prior knowledge, attitudes and beliefs about screening may have differed from those of non-users. However, the high levels of knowledge we observed, and relatively low levels of decisional conflict and worry, suggest that *BSD* did not have a detrimental impact on these important outcomes. Similarly, we did not find a large difference between the proportion of users with evidence of prior screening (74%), and the proportion of users who reported actual or intended use of screening mammography following their post-*BSD* preventive care visit (77%), suggesting that *BSD* was not biased for or against any particular screening strategy and did not cause dramatic changes in behavior. *BSD* did not appear to influence clinician perceptions of patient knowledge, but perhaps more importantly, it did not significantly increase clinician-perceived patient anxiety about screening or the length of discussions about screening. These results suggest that *BSD* did not add a burden to the clinical encounter or adversely affect the clinician-patient interaction from the provider perspective.

Our study included women of different racial and ethnic backgrounds, who also likely varied in their socioeconomic status. However, 80% of the sample was white, 77% were married, and 96% had commercial health insurance, and our findings may not be generalizable to different populations, particularly those with a greater proportion of African-American women or a greater proportion of uninsured women. Similarly, the numeracy, health literacy and internet comfort of our sample may not be generalizable to all US women in their 40s. Socially disadvantaged groups, including those with limited education or low literacy, may benefit from decision aids specifically tailored to their needs [28].

*BSD* itself has several limitations, some due to limitations of available evidence. For example, breast cancer risk estimates were personalized, but estimates of screening benefit were age group-specific. Other limitations reflect deliberate decisions to maximize user comprehension and reduce confusion. For example, breast cancer risk and screening benefit are presented as point estimates without ranges or confidence intervals. Effectively communicating uncertainty with lay audiences is challenging, and there is currently no consensus on best practices [29]. Although we reviewed many decision aids for cancer screening as we developed *BSD*, we cannot say whether *BSD* is superior to other web-based decision aids for breast cancer screening or even if a web-based tool is appropriate for all women in their 40s at low-to-average risk of breast cancer.

There are approximately 22 million women ages 40–49 in the US, each of whom is advised, by the USPSTF and others, to make an individualized decision regarding screening mammography in consultation with her physician. While some expert groups continue to recommend that women ages 40–49 be offered annual mammography, not all women accept this offer. Use of a decision aid could alleviate medico-legal concerns that some clinicians have in supporting their patients’ individualized screening mammography decisions, especially amid conflicting and often sensationalized reports in the popular press [30]. Even ACOG, which recommends annual screening mammograms, recently advised that decisions about screening start age and frequency “should be made through shared decision making,” and that “health care providers should work with patients to determine the best screening strategy based on individual risk and values” [31]. *BSD* directly addresses this recommendation, offering women personalized estimates of their breast cancer risk and an opportunity to explore their values and preferences. *BSD* also provides balanced, evidence-based information about screening options, consistent with recent calls for patient-centered, informed consent that meets a “reasonable patient” standard and advances shared decision making [32].

## Conclusions

In a large pilot study, users of *Breast Screening Decisions*, a web-based decision aid, reported high levels of knowledge about breast cancer risk and screening and low levels of decisional conflict and worry. Most users subsequently discussed breast cancer screening with a clinician, and *BSD* did not appear to adversely affect the patient-clinician interaction from the clinician’s perspective. Our results suggest that decision aids like *BSD* may help women in their 40s make informed, personalized decisions about screening mammography.

## Additional file

Additional file 1: Table S1. IPDAS Criteria. The supplemental table shows how *Breast Screening Decisions* meets the content and development criteria in the International Patient Decision Aid Standards (IPDAS) checklist. (PDF 74 kb)

## Abbreviations

ACOG: American Congress of Obstetricians and Gynecologists
BCWS: Breast cancer worry scale
*BSD*: *Breast Screening Decisions*
DCS: Decisional conflict scale
EHR: Electronic health record
IPDAS: International patient decision aid standards
USPSTF: US Preventive Services Task Force
