# Supplementary material for: Facilitating informed decisions about breast cancer screening: development and evaluation of a web-based decision aid for women in their 40s
Source: BMC Med Inform Decis Mak. 2017 Mar 21;17:29. doi: 10.1186/s12911-017-0423-7 (PMC5359988; doi:10.1186/s12911-017-0423-7)
Supplement: Additional file 1: Table S1. — IPDAS Criteria. The supplemental table shows how Breast Screening Decisions meets the content and development criteria in the International Patient Decision Aid Standards (IPDAS) checklist. (PDF 74 kb) [file 12911_2017_423_MOESM1_ESM.pdf]

Additional file 1: Table S1 - IPDAS criteria

**Content criteria: Does the decision aid…**	**Met fully**	**Met partially**
Provide information about options in sufficient detail for decision making?		
Describe the health condition	x	
List the options	x	
List the option of doing nothing	x	
Describe the natural course without options		x
Describe procedures	x	
Describe positive features [benefits]	x	
Describe negative features of options	x	
Include chances of pos/neg outcomes	x	
Describe what test is designed to measure	x	
Include changes of true pos, true neg, false pos, false neg results	x	
Describe possible next steps based on test result	x	
Include chances the disease is found out without screening	x	
Describe detection/treatment that would never have caused problems if one was not screened	x	
Present probabilities of outcomes in an unbiased and understandable way?		
Use event rates specifying the population and time period	x	
Compare outcome probabilities using the same denominator, time period, scale	x	
Describe uncertainty around probabilities	x	
Use visual diagrams	x	
Use multiple methods to view probabilities	x	
Allows the patient to select a way of viewing probabilities [words, numbers, diagrams]	x	
Allow patient to view probabilities based on their own situation [e.g. age]	x	
Place probabilities in context of other events		x
Use both positive and negative frames [e.g. showing both survival and death rates]	x	
Include methods for clarifying and expressing patients' values?		
Describe the procedures and outcomes to help patients imagine what it is like to experience their physical, emotional, social effects	x	
Ask patients to consider which positive and negative features matter most	x	
Suggest ways for patients to share what matters most with others	x	
Include structures guidance in deliberation and communication?		
Provide steps to make a decision	x	
Suggest ways to talk about the decision with a health professional	x	
Include tools [worksheet, question list] to discuss options with others	x	

Development criteria: Does the decision aid…	Met fully	Met partially
Present information in a balanced manner?		
Able to compare positive/negative features of options	x	
Shows negative/positive features with equal detail [fonts, order, display of statistics]	x	
Have a systematic development process?		
Includes developers' credentials/qualifications	x	
Finds out what users [patients, practitioners] need to discuss options	x	
Has peer review by patient/professional experts not involved in development and field testing	x	
Is field tested with users (patients facing the decision; practitioners presenting options	x	
The field tests with users [patients, practitioners] show the decision aid is:		
Acceptable	x	
Balanced for undecided patients	x	
Understood by those with limited reading skills (not target group)		x
Use up to date scientific evidence that is cited in a reference section or technical document?		
Provides references to evidence used	x	
Report steps to find, appraise, summarize evidence		x
Report date of last update	x	
Report how often patient decision aid is updated	x	
Describe quality of scientific evidence [including lack of evidence]	x	
Uses evidence from studies of patients similar to those of target audience	x	
Disclose conflicts of interest?		
Report source of funding to develop and distribute patient decision aid	x	
Report whether authors or their affiliations stand to gain or lose by choices patients make after using the decision aid	x	
Use plain language?		
Is written at a level that can be understood by the majority of patients in the target group	x	
Is written at a grade 8 equivalent level or less according to readability score [SMOG or FRY]	x	
Provides ways to help patients understand information other than reading [audio, video, in-person discussion]		x
